# Physician assistants in geriatric medical care

**DOI:** 10.1186/s12877-020-01831-1

**Published:** 2020-11-04

**Authors:** Andrzej Kozikowski, Trenton Honda, Freddi Segal-Gidan, Roderick S. Hooker

**Affiliations:** 1 National Commission on Certification of Physician Assistants, Johns Creek, GA 30097 USA; 2 Northeastern University, 202 Robinson Hall, 360 Huntington Avenue, Boston, MA 02115 USA; 3 Departments of Neurology and Family Medicine, University of Southern California (USC) Keck School of Medicine, Los Angeles, CA USA; 4 Neurobehavior & Alzheimer’s Center and Rancho/USC California, Alzheimer’s Disease Center at Rancho Los Amigos National, Rehabilitation Center in Downey, Downey, CA USA; 5 Northern Arizona University, 15917 NE Union Rd, Ridgeview, WA 98642 USA

**Keywords:** NCCPA, Physician associate, Gerontology, Geriatrics, Medical economics, Nurse practitioners

## Abstract

**Background:**

The US population is maturing. As of 2020, over 52 million (16%) people are age 65 or older. With a citizenry that is increasingly “gray,” the nation is short of medical providers who specialize in geriatric medical care. For example, the number of geriatrician physicians per 10,000 adults 65 years and older has decreased since 2000, with approximately 5300 in 2018. Nurse practitioners in geriatric medical care numbered 598 in 2018. Considering that the projected needs by 2030 will be over 30,000, the trajectory of geriatricians is becoming increasingly inadequate for the aging population. Physician assistants (PA) are another class of providers that are filling this geriatric medical care role, although little has been published. To address this role of PAs a study was undertaken.

**Methods:**

The National Commission on Certification of Physician Assistants databank provided the number and characteristics of PAs in geriatric medicine and compared them to all other certified PAs. Analyses included descriptive statistics, Chi-Square, and Wilcoxon Rank Sum tests for comparisons between PAs practicing in geriatric medical care vs. all other PA specialties. Where a comparison between PAs in geriatrics and other specialties was made, a *P* value of .05 or less was considered statistically significant.

**Results:**

As of 2018, there were 794 certified PAs, or 0.8% of the certified PA workforce, in geriatric medical care. This cadre has grown significantly since 2013, both in total number (increasing over 373%) and as a percentage of the PA workforce. The median age of certified PAs in geriatrics is 45 years, and 79% are female. Almost half (46%) of PAs in geriatric medicine work in extended care facilities or nursing homes, which differs from PAs in non-geriatric care. Another 8% work in government facilities and 8% in rehabilitation facilities. In 2018, the mean annual income for this PA group was $106,680.

**Conclusions:**

As the American population continues to age, the relative growth of PAs in geriatric medicine makes them a natural part of the solution to the projected physician geriatrician deficit. The role of PAs in geriatric medical care remains to be explored.

## Background

Increased life expectancy and declining birth rates are changing the demographics of America. At the end of the second decade of the twenty-first century, over 52 million people are age 65 or older, making up 16% of the population [1]. According to the US Census Bureau, by 2030 all baby boomers will be 65 or older. This will enlarge the older adult population such that one in every five will be “retirement age” [1]. With a population that is increasingly older the nation faces a shortage of medical providers who specialize in geriatric medical care. The number of geriatrician physicians per 10,000 adults older than 65 years has decreased steadily since 2000, and the total geriatrician workforce number was approximately 5300 as of 2018 [2]. Nurse practitioners (NPs) in geriatric care are an order of magnitude smaller, numbering 598 in 2018 [3]. Considering that the projected geriatricians needed by 2030 will be over 30,000, the trajectory of medical providers will become increasingly inadequate for the aging population [4].

To address this “geriatric imperative,” greater demands are placed on medical educators to mentor and clinically train clinicians who can meet the needs of the aging population. The National Academy of Medicine recommends that comprehensive humanistic medical education in geriatrics be integrated throughout the curricula of medical schools along with physician assistant (PA) and NP education programs [2]. Based on their broad-based primary care medical training, PAs are ideally situated to help meet this increasing shortfall, but there is limited information on the trajectory and practice characteristics of the PA geriatric workforce. To address the limited literature on the subject, we set out to build a profile of PAs in geriatric medicine. The aim was to describe the stock of PAs in geriatric medical care and set the stage for needed investigation essential to inform research, clinical managers, medical workforce analysts, and policymakers about this overlooked medical workforce.

## Method

This study draws on data collected by the National Commission on Certification of Physician Assistants (NCCPA). These data contain specific demographic and self-reported practice information on all certified PAs in the US [5]. NCCPA developed an online data collection tool, the *PA Professional Profile*, to efficiently gather PA health workforce data. Certified PAs regularly update the data collected via the *PA Professional Profile* as they access the secure online portal or when they log Continuing Medical Education activity. Reminders are provided to PAs who have not refreshed their profile in the last three years. The *PA Professional Profile* is an optional algorithm-driven survey consisting of a set of questions about the type and characteristics of the practice where the PA is employed [6].

Data for this study were extracted from the *PA Professional Profile* and NCCPA’s database. The variables in the profile instrument are standard variables and remain mostly unchanged since being launched in 2012; however, a few new variables are added yearly. In addition to demographics such as age, gender, and state where licensed, the following were the *PA Professional Profile* questions used in the present study:

- *Which of the following best describes your principal area of clinical practice?* Response options were medical and surgical specialties and sub-specialties.
- *Which of the following best describes the type of practice setting in which your principal clinical PA position is located?* Response options included a hospital, extended care/nursing home, office-based private practice, etc.
- *Please estimate your total income before taxes from January–December of the last calendar year from all of your PA positions combined.*

Of the total number of certified PAs at the end of 2018 (*n* = 131,152), excluded were those who **a**) did not update their NCCPA profile in the last 3 years, **b**) indicated they were not active clinically, or c) did not answer the practice specialty question. These criteria resulted in the exclusion of 24.8% of all certified PAs, for a final study population of 98,625. Data were extracted on the number of PAs in geriatric medical care, their distribution, income, and how they compare to certified PAs in all other specialties. Analysis included descriptive statistics as well as Chi-Square and Wilcoxon Rank Sum tests, as appropriate, for demographic and practice characteristics comparisons between PAs practicing in geriatrics vs. all other clinical specialties. For all analyses where a comparison between PAs in geriatrics and other specialties was made, a *P* value of .05 or less was considered statistically significant. Statistical analyses were conducted using R (R Core Team 2020; R: A language and environment for statistical computing; R Foundation for Statistical Computing, Vienna, Austria - URL https://www.R-project.org/).

## Results

**Fig. 1:**
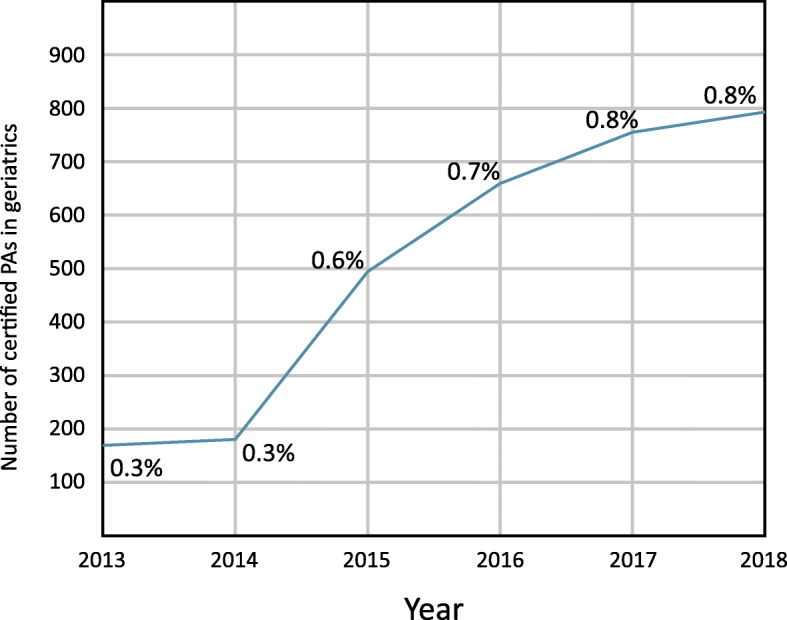
Growth of PAs in Geriatric Medicine

At year’s end 2018, 794 certified PAs in the US self-identified as working in geriatric medical care, representing 0.8% of the study population (*n* = 98,625). Of note, the proportion of PAs working in geriatric medicine has grown by over 167% since 2013, while the absolute number of PAs working in geriatric medical care has increased by 373% (Fig. 1).

**Fig. 2:**
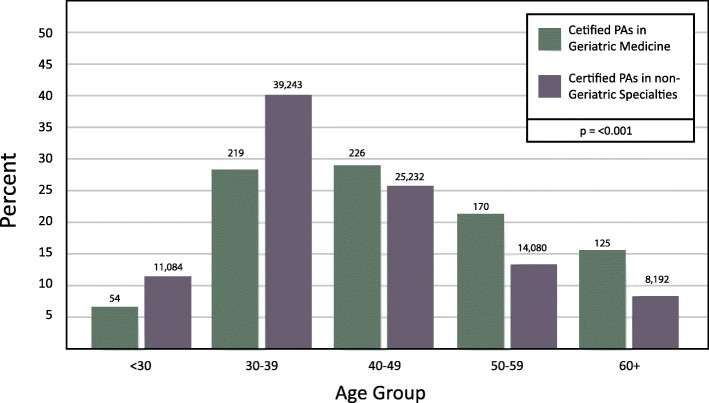
Age of PAs in Geriatric Medicine vs. non-Geriatric Medicine Specialties (2018)

The PA geriatric medicine workforce is, on average, older than PAs practicing in disciplines other than geriatric medicine (median age 45 versus 39, respectively). Over 62% of PAs working in geriatric medical care are < 49 years old (Fig. 2). However, PAs in geriatric medicine are underrepresented among the youngest PA age band compared to PAs working in all other specialties (< 30 years: 6.8% PAs in geriatric medicine versus 11.3% non-geriatric medical specialties), and overrepresented among the oldest PA age group (60+ years: 15.7% PAs in geriatric medicine versus 8.4% non-geriatrician PAs - Chi-Square = 124.966, DF = 4, *p* < 0.001).

**Fig. 3:**
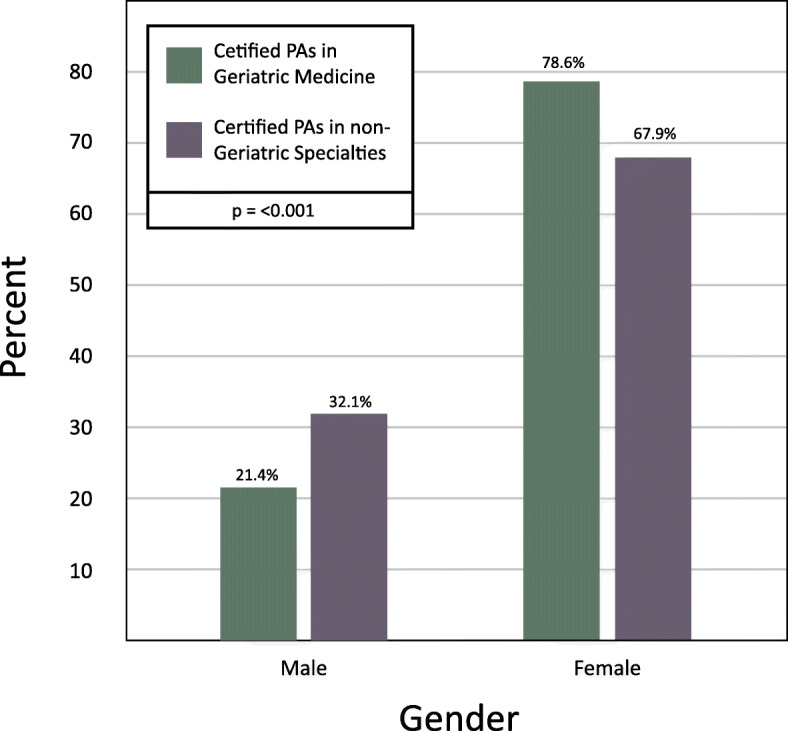
Gender of PAs in Geriatric Medicine vs. non-Geriatric Medicine (2018)

The PA geriatric medical care workforce is composed of a higher percentage of females than the remainder of the PA workforce. Approximately 79% of PAs working in geriatric medicine are female as compared to 68% of PAs in non-geriatric medical care specialties (Fig. 3) (Chi-Square = 41.580, DF = 1, *p* < 0.001).

When comparing years of practice between certified PAs in geriatric medicine and all certified PAs, on average, PAs working in geriatric medicine tend to have been in practice longer (Table 1). Among recently certified PAs (1–5 years), the percent working in geriatric medicine is less than that of all other specialties (18.3% vs. 28.6%, respectively) (Chi-Square = 81.780, DF = 8, *p* < 0.001). Moreover, PAs working in geriatric medicine have, on average, been certified and practicing longer (W = 46,101,336, *p* < 0.001); and for those PAs certified over 15 years, there is a significantly larger percent practicing in geriatric medicine relative to all other specialties (Table 1).

**Table 1:** Years since certified –PAs in geriatrics versus non-geriatric specialties 2018

Years certified	Certified PAs in geriatrics		PAs not in-geriatric medicine		
	Number	Percent	Number	Percent	*p*-value
1–5	145	18.3	27,929	28.6	< 0.001
6–10	173	21.8	23,620	24.2	
11–15	141	17.8	17,378	17.8	
16–20	150	18.9	14,197	14.5	
21–25	89	11.2	7651	7.8	
26–30	41	5.2	2903	3.0	
31–35	31	3.9	2329	2.4	
36–40	17	2.1	1399	1.4	
> 40	7	0.8	357	0.4	
Mean (SD)	14.6 (9.20)		11.9 (8.58)		< 0.001
Median	13		10		

**Fig. 4:**
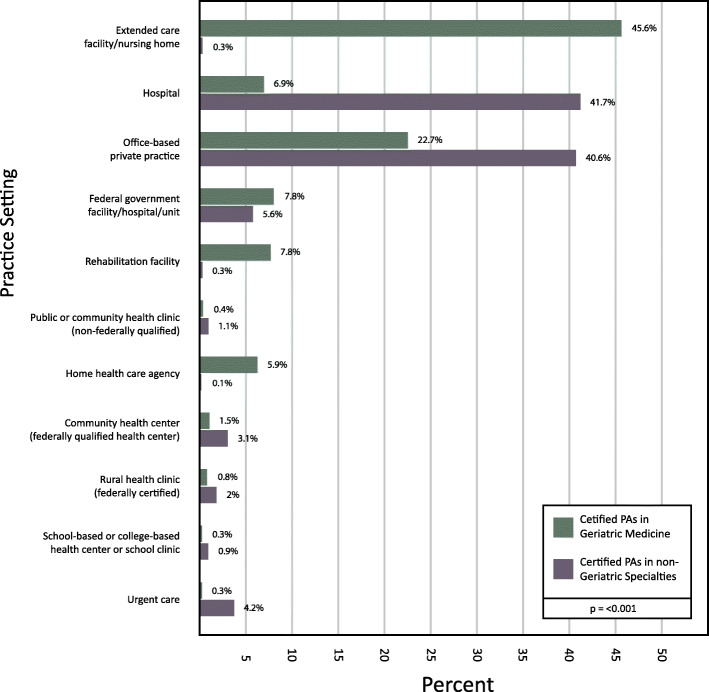
Practice Setting of Geriatric PAs vs. Non-Geriatrics Sepcialties (2018)

The principal clinical practice setting of PAs employed in geriatric medicine differs substantially from PAs working outside of geriatric medicine as a specialty (Fig. 4). Almost half (46%) of PAs who report working in geriatric medicine identify their primary location of employment as extended care facilities or nursing homes, while less than 1% (0.3%) of PAs in non-geriatric specialties work in such settings. Another 23% are office-based in private practices, which is less than the 41% of PAs in all other specialties who self-report this employment setting. Additionally, 8% of PAs in geriatric medicine work in federal government facilities (such as the Department of Veterans Affairs or a federal prison), and an additional 8% of PAs in geriatric medicine work in rehabilitation facilities (Chi-Square = 25,906.57, DF = 10, *p* < 0.001).

**Fig. 5:**
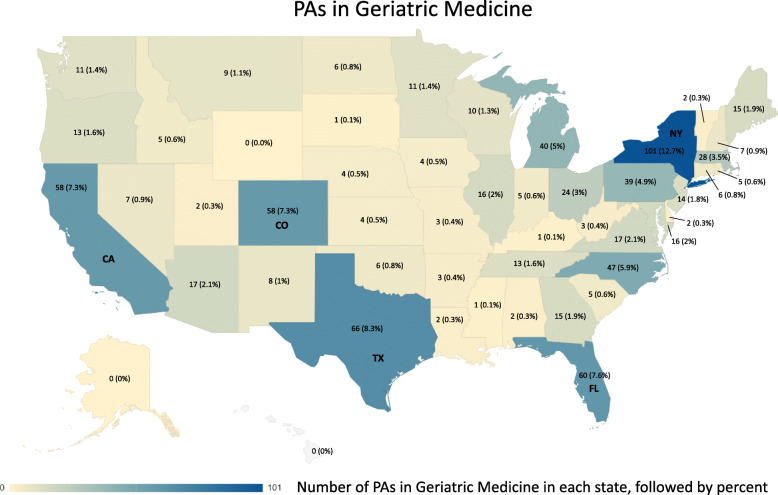
Geographic Distribution of PAs in Geriatric Medicine (2018)

The distribution of PAs in geriatric medical practice by state is shown in Fig. 5. The five states with the largest numbers of geriatric medical PAs are – Florida, California, Colorado, New York, and Texas.

**Fig. 6:**
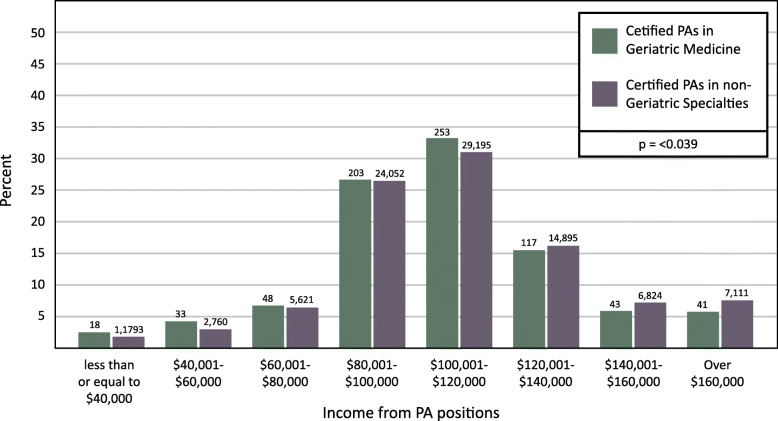
Income of PAs in Geriatric Medicine vs. Non-Geriatric Medicine Specialties (2018)

Self-reported income from PAs in geriatric medicine was compared to PAs in all other specialties (Fig. 6). The mean income of PAs in geriatric medicine was $106,680, while the median was $105,000. For PAs in all other specialties, the mean and median were $111,073 and $105,000, respectively. PAs working in geriatric medicine were more likely to earn between $100,001 - $120,000, compared to those in all other specialties (Chi-Square = 14.751, DF = 7, *p* = 0.039).

## Discussion

To the best of our knowledge, this is the first study to describe the demographic and occupational profile of PAs working in geriatric medicine. At year’s end 2018, 0.8% of certified PAs were practicing in geriatric medicine, with significant growth (167%) in this specialty practice area over a six-year span. During this time, the absolute number of PAs practicing in geriatric medical care grew by 373%. The median age of the 2018 PA cohort practicing geriatric medicine is 45 years, and 79% are female.

The trajectory of PA growth in geriatric medicine is in an acceleration phase, suggesting that this medical specialty is in strong demand. This growth is not matched by physicians and nurse practitioners [3, 4]. For example, the physician geriatrician workforce is anticipated to grow just 17.5% by 2025, a total of 6230, with a projected deficit of physician geriatrician providers of 26,980 by 2025 [4]. Nor is it matched by change in the PA workforce during this same time period, which is projected to increase by 31% (2019–2029) [7]. Not unlike physician geriatricians, the majority of PAs who work with older adults are female [5]. Moreover, PAs in geriatric medicine practice in states with the highest proportion of older adults such as Florida, California and Texas [7].

An important practice distinction for PAs in geriatric medicine is their presence in long term care facilities – almost half (46%) identify working in an extended care or nursing home facility [8]. Less than 1 % of PAs in all other specialties list their practice setting as a nursing home or comparable care institution. Another 8% work in rehabilitation localities, compared to < 1% of PAs in non-geriatric medical specialties. This is similar to the NP workforce in geriatric medical care, which is concentrated in long term care facilities but smaller in number than PAs in geriatric medicine [3].

Knowing the number and characteristics of the PA geriatric medical workforce is the first step in taking stock of how medical care is being delivered to an aging American population by a cadre of medical providers. Such medical provider information is needed in planning for a society with a growing number and proportion of older adults, as well as the characteristics of those focused on the care of the aged [9]. This new knowledge holds implications for health workforce planning, deployment projections, policymaking, and estimating numbers of physicians [10].

In summary, the American physician assistant movement is in a growth phase, and geriatric medicine is one of the 70 medical and surgical roles where they are represented [5]. Their numbers in this medical specialty are significant, and their employment settings suggest this is where high concentrations of older adults are located.

### Limitations and strengths

This study draws upon data from NCCPA’s *PA Professional Profile*, which is the most comprehensive national collection of workforce data on PAs [11]. The use of self-report data is always subject to misinterpretation of the question and the option of not completing the algorithm-driven questionnaire embedded in the NCCPA secure portal [12]. However, survey participation and validation attestation research suggest the NCCPA data are reliable and overlap well using federal data comparisons [13]. This authentication is reassuring that the reported results are valid and representative. In this presentation we sought to provide a descriptive overview of the current state of PAs in geriatric medicine. Future research could expand on our descriptive study by adjusting for covariates, and/or triangulate these findings with national data rooted in state and federal agencies, such as the US Census, Bureau of Labor Statistics, American Community Survey, and others.

## Conclusion

Understanding PA characteristics and employment settings is an important footing in how this profession is responding to medical labor supply and demand forces. In this undertaking, a six-year trend analysis revealed that the percent of the PA workforce in geriatric medicine is growing substantially and represents a needed source of expertise in American medical care delivery. That the majority are working in extended care facilities and private physician offices suggests they are deployed where the older adult medical interface is occurring. With this foundation of the contemporary PA geriatric medical workforce, the next step is to understand the economics of such labor, outcomes of care, relationships with other members of the medical team, and patient satisfaction.

## Data Availability

The data that support the findings of this study are available from the NCCPA. Some restrictions may apply to the availability of these data, which were used under license for the current study. A minimal dataset (anonymous to individuals) is available for analysis and was used during the study.

## Abbreviations

CA: California
CO: Colorado
FL: Florida
NCCPA: National Commission on Certification of Physician Assistants
NP: Nurse practitioner
NY: New York
PA: Physician assistant or physician associate
TX: Texas
US: United States of America
